# Medical Reform

**Published:** 1845-04

**Authors:** 


### MEDICAL REFORM.

Remarks on the Tripartite Division of Medicine. By *Michael Keating O'Shea.* Gilbert, 1845.

A Bill for Regulating the Profession of Physic and Surgery. (Ordered to be Printed, Feb. 1845.)

The Apothecaries' Society's (2nd) Address to the General Practitioners on the New Medical Bill, as contrasted with the Bill of last Session. Highley, 1845.

Being among those who refused to join in an indiscriminate opposition to the former measure proposed by Sir James Graham, in the hope and belief that, when informed of the wishes and just expectations of the Profession (whose opinion he stated himself so anxious to be placed in possession of), that Minister would consent to such alterations and amendments as would render it a useful and satisfactory enactment, we deeply regret having to record the disappointment the perusal of the provisions of the new Bill has occasioned us. The General Practitioners neither can or ought to feel contented with this reply to their just and reasonable demands. It is true the new measure was ushered in with a conciliatory speech on the part of its proposer, and a distinct recognition of the usefulness, importance, honorable conduct, and humane dispositions of this large class of society. But as we never believed that the former measure was introduced, as roundly stated by so many of its opponents, with the express intention of degrading the great mass of the Profession, so we are quite at a loss to perceive how the value of the present one is enhanced by an admission which we suppose any other member of the House of Commons would have made just as cheerfully. On the contrary, we see cause for discouragement in this. If Sir James Graham, while acknowledging the excellent qualities and vast usefulness of the General Practitioner, still witholds from him his most elementary rights, we can only come to the conclusion that he remains, after all the pains which have been taken to instruct him, hopelessly ignorant of what these are, or is prevented by some influence adverse to their recognition from doing justice to them. That the former element is not an inconsiderable one in determining his proceedings, we may judge from his astounding reply to a question asked him recently in the House, as to his intentions in regard to amending the Charter granted to the College of Surgeons. He stated, he was "at a loss to understand what were the grievances or exclusions his hon. friend's question referred to"! This, joined with the belief expressed in his speech that the good understanding between the governing powers and members of the College would shortly become augmented by the operation of this very charter, shews such a state of utter ignorance upon the very matter which has most excited the attention and dissatisfaction of the whole profession, that the expectation of its removal must be entertained by those only who are in possession of more sanguine temperaments than ourselves.

It is true the Minister stated he thought the General Practitioners ought to have a voice in the deliberations of the Council of Health; and that one or two of his nominees would be taken from their body. But this is not sufficient. A much larger proportion should be admitted, and such admission should not depend on the caprice of the Home Secretary of the day, but be as definitely provided for as is that of the representatives of the other branches of the Profession. Then, again, neither the Act itself or the Statement contain any provision for, or allusion to what is, after all, the great object for which all medical reformers have been struggling, viz. *the complete representative system.* So far as the Charters hitherto, or about to be granted, are concerned, we find this, the main-spring of all future improvements in the governance of our various medical institutions, completely trodden under foot; and the surgeon, physician, and general practitioner, deliberately informed they are unable to perform elective functions entrusted to the pharmaceutist, the veterinarian, and the meanest burgess. It remains to be seen whether the Crown will consent to the more liberal construction of the now inevitable Charter of Incorporation of General Practitioners.

It is also true that Sir James Graham has given in to what he considers the prejudices of the profession as respects the punishment of unlicensed practitioners. But if ever a benefit was marred by the mode of its bestowal and the contingencies of its enjoyment, it is the case here. In the place of a simple penal clause directed against all unregistered persons, who engaged in the practice of physic, whether under the ordinary professional titles or no titles at all, we are to have the Apothecaries' Act continued in existence for the performance of that of its functions which of all it had been found least able to compass; and that too when the funds of the Society will have become much diminished by the loss of examination fees. It is true fines will be inflicted upon those who assume one of the recognized professional appellations; but how easily such a provision as this will be evaded need not be told.

It is but due to the Apothecaries' Society to admit they have seen as early as others the defects of the Act which regulated their proceedings, and have done what lay in their power to remedy these; and the preference they have shewn of merging themselves into an improved organization of General Practitioners, to constituting a mere subordinate fragment of the new Examining Board is a very proper and honorable one. No valid reason exists why their Act should not be forthwith abolished as originally contemplated. Its retention will only give rise to confusion, and prove an impediment to future progress.

If we understand the new Bill aright, the title of "Surgeon" is intended to be confined to those who have undergone a second special examination before the College of Surgeons, and will, in that case we suppose, be equivalent to "Fellow." If so, the provision is a most unjust one, and can only have a tendency to lower the great body of general practitioners in the eyes of the public. Always having hitherto borne this title, the forcing them to assume the name of mere Licentiate is neither more or less than a degradation. Another injustice contemplated in this Bill is the obliging a licentiate or fellow who has obtained his diploma in one part of the so-called United Kingdom, to pay additional fees for liberty to practise in another portion, which is under the jurisdiction of another College. He is allowed to practise without additional examination, but not without additional fees. A man found competent to practise in one part of the kingdom, and having paid for his diploma, should certainly not be taxed afresh in this way.

In the interval which has elapsed between the introduction of the two Bills, the Association of General Practitioners has made a most rapid advance—numbering now, we believe, very near four thousand members. The deplorable and suicidal policy followed by the Council of the College of Surgeons, which conceived in utter ignorance of the high spirit of the great body of its members, is adhered to with a foolish obstinacy; and the unsatisfactory extent of the changes proposed by Sir James Graham, holding out now so little probability that he will consent to such as will prove acceptable, have rendered this great organization absolutely essential, and so imposing. We perceive with pleasure that one great defect in the constitution of the Examining Board of the Apothecaries' Society is not intended to be persevered in, viz. the confining the Examiners to one grade of the Profession. Conjoined with general practitioners, there are to be men eminent in various branches of medical science, and experienced in teaching. This is as it should be, if the General Practitioner wishes to advance his position; and with a body so constituted we confidently anticipate finding the examinations become more practical in their character and more honorable to the successful candidates. We are not among those who anticipate any deterioration of their rank in public estimation, any loss of caste, will be sustained by the Incorporation of the General Practitioners into a separate body. On the contrary, we believe a separation from the drug-selling concerns of the Apothecaries' Society, and an improvement in the mode of examination, will be attended with a proportionate elevation of character and consideration. To submit to the existing and contemplated state of things would, on the other hand, insure and merit degradation.

Mr. O'Shea's "Remarks" contain nothing new upon the subject of Medical Reform, and are drawn up in a very rambling manner. The "Address" of the Society is a temperate examination of the provisions of the New Bill, which is rather more leniently dealt with than we should have expected from the tenor of the former publications of the Society. With the following extract we shall conclude this notice.

"A new clause has been introduced into the present Bill, empowering the Council of Health to erase from the register the name of any person who shall be convicted of felony, or who shall be found to have procured his registry by fraud, or to have wilfully given a false certificate in any case in which the certificate of a Physician or Surgeon is required by law; and this punishment is to be followed by the total loss of all the privileges of a Medical Practitioner. The Society would suggest, that this clause would be more likely to effect its own object if a power were given to the Council of Health, to restore a person to the Register whose name had been erased, if a fitting case for the exercise of such a power should present itself. The inability of the Council, *under any circumstances*, to restore a name which has once been erased from the Register, would probably be found to render the Council unwilling to exercise its power in cases which, under other circumstances, would justly merit such a punishment."

